# Wheat Meal vs. White Flour

**Published:** 1890-03

**Authors:** S. B. Childs


### WHEAT MEAL VERSUS WHITE FLOUR.

BY S. B. CHILDS, M. D.

To attain a condition of perfect health certain requirements must be fully met. This fundamental declaration is imperative, and the disobeying of the law is constantly showing itself in a diversity of ailments.

The various forms of machinery that are used in our manufactories receive constant attention. Only the kinds of oil are applied that have the best lubricating power, and as a result, the machinery runs smoothly and even noiselessly. The human machine *per contra* is constantly getting out of order, the nerves, bones and muscles are imperfectly supplied with the proper pabulum, and as a consequence, we have disturbances manifesting themselves in the shape of disordered health. The blood cries out, when its victim cringes with neuralgic pains, for better sustenance, but the cry is generally in vain,—the same food is supplied *ad nauseam.* Why should men heed the laws that govern the inanimate, and not pay due attention to the living organism ? Perhaps we find the key to this heedlessness in the words of that great stoic philosopher Seneca, who wrote eighteen hundred years ago: "There is nothing against which we ought to be more on guard, than like a flock, following the crowd of those who have preceded us,—going as we do, not where we ought to go, but where men have walked before."

Draper in his physiology likens the body to the flame of a candle,—it is constantly being fed and is constantly burning away. The quality of the light will wholly depend on the material supplied. In the same way, to attain a robust, vigorous, and healthy condition, mankind must take as food only that sort which contains those ingredients that are essential. Too much of one kind, or too little of another, disturbs the equilibrium. Bread has been called the staff of life, and yet this figure of speech, in view of the kind of bread that most persons eat, is a decided misnomer. The ordinary white flour, which forms the basis of so much food that is eaten, is principally a starch compound, and contains only three of the fifteen elements that go to compose the body, namely, carbon, hydrogen, and oxygen.

To prove that white flour does not meet the requirements of the body, Magendie fed it wholly to a number of dogs, and at the end of forty days they died. Others to whom he gave the wheat meal, at the end of this time were in first-class condition. More than half of the children under twelve years of age have decayed teeth, owing to the insufficient supply of the required mineral ingredients, and this deficiency is caused as a rule by eating white bread. Dyspepsia, constipation, loss of nerve power, and many other diseases are produced by improper feeding. Sulphur is required for the growth of the hair, yet white flour does not contain a trace ; the phosphates are also notably lacking, and as these substances are absolutely necessary in the animal economy, then, arguing *a priori*, the use of bread as ordinarily prepared should be interdicted.

When flour is made of the whole grain of the wheat, we have an article of food which contains all the elements that the body requires for its support; and this flour should be universally used in spite of the false æsthetic taste that demands a "white loaf"; for such theory of taste tested by the canons of common-sense loses its force; and what the body demands should be the touch-stone, rather than what pleases the eye of the unthinking housewife.

To paraphrase the words of Sydney Smith, in reference to the various forms of errors that still hold with tenacious grip their sway: "The centuries that have passed have had ample opportunity to display the full bloom of their imbecility," and it would seem quite time to call a halt in the way of improper feeding.

Sir Henry Thompson in an article on "Diet," says: "I have come to the conclusion that a proportion amounting at least to more than one-half of the diseases which embitter the middle and latter part of life, among the different classes of the population, are due to avoidable errors in diet.

